# The role of ATP signalling in response to mechanical stimulation studied in T24 cells using new microphysiological tools

**DOI:** 10.1111/jcmm.13520

**Published:** 2018-02-01

**Authors:** Na N. Guan, Nimish Sharma, Katarina Hallén‐Grufman, Edwin W. H. Jager, Karl Svennersten

**Affiliations:** ^1^ Department of Molecular Medicine and Surgery Section of Urology Karolinska Institutet Stockholm Sweden; ^2^ Department of Urology Karolinska University Hospital Stockholm Sweden; ^3^ Department of Physiology and Pharmacology Karolinska Institutet Stockholm Sweden; ^4^ Department of Physics, Chemistry and Biology (IFM) Linköping University Linköping Sweden

**Keywords:** ATP, Ca^2+^ signalling, mechanostimulation, microphysiological systems, organ on chip, P2X, P2Y

## Abstract

The capacity to store urine and initiate voiding is a valued characteristic of the human urinary bladder. To maintain this feature, it is necessary that the bladder can sense when it is full and when it is time to void. The bladder has a specialized epithelium called urothelium that is believed to be important for its sensory function. It has been suggested that autocrine ATP signalling contributes to this sensory function of the urothelium. There is well‐established evidence that ATP is released via vesicular exocytosis as well as by pannexin hemichannels upon mechanical stimulation. However, there are still many details that need elucidation and therefore there is a need for the development of new tools to further explore this fascinating field. In this work, we use new microphysiological systems to study mechanostimulation at a cellular level: a mechanostimulation microchip and a silicone‐based cell stretcher. Using these tools, we show that ATP is released upon cell stretching and that extracellular ATP contributes to a major part of Ca^2+^ signalling induced by stretching in T24 cells. These results contribute to the increasing body of evidence for ATP signalling as an important component for the sensory function of urothelial cells. This encourages the development of drugs targeting P2 receptors to relieve suffering from overactive bladder disorder and incontinence.

## 1 INTRODUCTION

Dysfunction of the urinary bladder such as overactive bladder syndrome (OAB) is a common and stigmatizing problem. In OAB, the patient suffers from a disabling increase in micturition frequency and nocturia.^1^ The prevalence of OAB is 11%‐19%, and the current options for pharmacological treatment are often associated with lack of efficiency or poor tolerability.^2^ This condition impacts the quality of life of many patients worldwide, thus deserving further attention. Understanding the underlying molecular mechanisms is of outmost importance to help find new pharmacological treatments.

The current drugs to treat OAB are targeted towards cholinergic and adrenergic receptors; however, the contribution of purinergic nucleotides in bladder physiology was already described in 1972.^3^ Purinergic mechanosensory transduction is a well‐known mechanism by which information about mechanical stress is relayed to the central nervous system via nerves expressing purinergic receptors.^4^ This mechanism is of importance for urinary bladder physiology, which was clearly demonstrated in key studies using P2X3 knockout mice.^5^, ^6^ The significance of P2X receptors and purinergic mechanosensory transduction in the urinary bladder was further established by Rong et al in a study describing P2X activation in low and high threshold afferent nerve fibres in the urinary bladders of mice.^7^

A central component of purinergic mechanosensory transduction is the release of ATP from the affected cell or tissue as a result of mechanical stimulation. The release of ATP from urothelial cells upon mechanical stimulation has been described in a number of reports.^8^, ^9^, ^10^ This has also been seen in erythrocytes as well as in endothelia.^11^, ^12^, ^13^, ^14^ The current view of ATP in the voiding reflex can be summarized as follows: Distension of the urothelium triggers release of ATP which acts on P2X3 receptors on afferent sensory nerves in the suburothelium contributing to the sensation of bladder fullness. Efferent parasympathetic nerves innervate the detrusor smooth muscle. There, ATP is released as a co‐transmitter together with acetylcholine. ATP acts on P2X1 receptors on smooth muscle and contribute to the initiation of contraction. In the healthy human bladder, the purinergic contribution is only about 5% of the parasympathetic nerve‐mediated contraction, but this fraction is known to increase to about 40% in pathological bladder conditions.^15^, ^16^, ^17^, ^18^ Of importance for the findings presented in this report are previous studies that have shown an increase in ATP in the urine from patients with OAB and bladder obstruction due to prostate hyperplasia.^19^, ^20^

There are several comprehensive reviews available discussing the current knowledge of purinergic signalling in the urinary bladder.^21^, ^22^, ^23^ Yet, purinergic signalling in the urinary bladder still has many aspects to be explored. For example, the urothelium itself, in addition to its ability to release ATP, also expresses both ionotropic P2X and metabotropic P2Y purinergic receptors.^24^, ^25^, ^26^, ^27^, ^28^ This opens for the possibility that mechanical stimulation of the urothelium can initiate autocrine purinergic signalling, which in turn could contribute to cellular mechanotransduction. Cellular mechanotransduction, in this case, defined as the capacity of living cells to sense and integrate external mechanical stimuli and to convert them to intracellular signals.^29^

**Figure 1:**
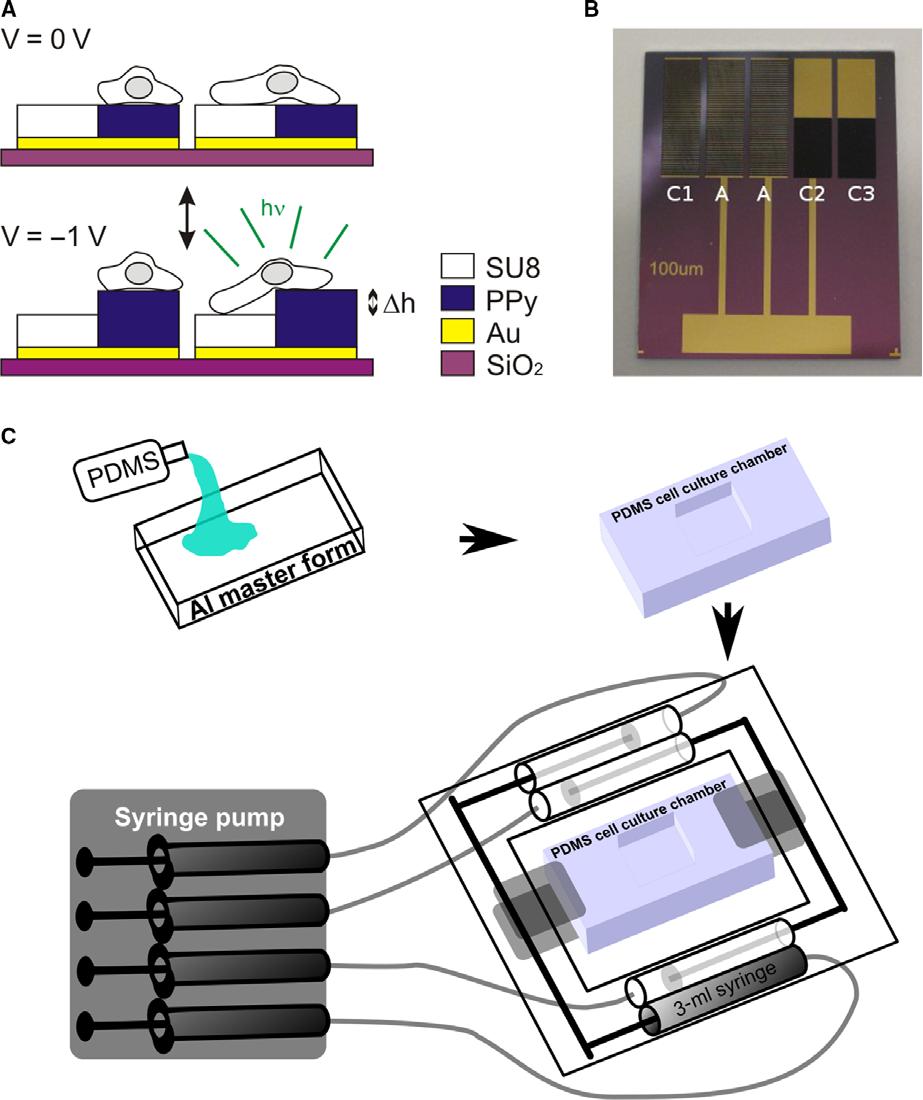
Microphysiological systems. A, Illustration of the principle of cell stretching by polypyrrole (PPy) microactuators. Upon application of a potential, the PPy microactuator will expand vertically stretching cells that are situated on the borders, that is that adhere to the surface of both PPy and the passive polymer SU8. Cells that are located on the surface of only PPy or the passive polymer SU8 are not mechanically stimulated. B, Photograph showing mechanostimulation chip 2 × 2.5 cm^2^ comprising two actuator areas indicated by A and three integrated areas for control experiments C1‐C3. The actuator areas comprise arrays of 100‐μm‐wide PPy actuators and 100‐μm‐wide SU8 lines. Panels A and B are modified from Svennersten et al^31^ C, Schematic depicting the process of manufacturing and addressing the cell stretch chamber. Polydimethylsiloxane (PDMS) is the silicone elastomer used for manufacturing of the cell stretch chamber

In this study, we investigated the changes in intracellular Ca^2+^ concentration induced in T24 urothelial cells by mechanical as well as direct ATP stimulation. We tested the hypothesis that ATP contributes to the Ca^2+^ response induced by mechanical stimulation in T24 urothelial cells. To accomplish this, new microphysiological systems^30^ for mechanical stimulation of cells in culture were introduced: a microfabricated chip, which allows mechanical stimulation and *in situ* recordings of the intracellular Ca^2+^ concentration in single cells, as well as a silicon‐based stretch chamber for mechanical stimulation of a population of cells^31^ (Figure 1).

## 2 MATERIALS AND METHODS

### 2.1 Cell culture

The bladder cancer cell line T24 (ATCC, no. HTB‐4) was propagated in Dulbecco's modified Eagle medium (DMEM) (Gibco) supplemented with 10% foetal bovine serum (FBS) (Gibco), 1% GlutaMAX (100X) (Gibco) and penicillin; streptomycin (100 U/mL; 100 mg/mL, Sigma‐Aldrich). Cells were detached from the cell‐culturing flask with 0.025%‐Trypsin‐EDTA (Gibco) in CaCl_2_/MgCl_2_‐free Dulbecco's phosphate‐buffered saline (DPBS) solution and washed once in DMEM, before 3 mL of cell suspension (1.0‐3.0 × 10^5^ cells/mL) was added to 30‐mm cell culture dishes (Sarstedt) with or without microactuator devices. Cells were incubated in a humidified 37°C, 5% CO_2_ cell incubator. The cell line was checked against the ICLAC Database of Cross‐Contaminated or Misidentified Cell Lines version 7.2. Cell culture was regularly screened for mycoplasma contamination by DNA staining using Hoechst 33258.

### 2.2 Calcium imaging

Loading of cells with Fura‐2 was performed during 40‐minutes incubation at room temperature in DMEM/F12 (Gibco) with 2 μmol L^−1^ Fura‐2‐AM (Life technologies) and 0.03% Pluronic F127 (Sigma‐Aldrich). Samples were mounted on a Nikon upright Eclipse 80i microscope with a Nikon Fluor 20X/0.5W dip down objective. Excitation at 340 and 380 nm was achieved with a DeltaRAM illuminator and a DeltaRAM‐V monochromator with a computer‐controlled SC‐500 shutter controller. Emissions (510 nm) were collected using a Photometrics Coolsnap CCD camera from Roper Scientific. Data were analysed with Image J (U. S. National Institutes of Health).

### 2.3 Mechanostimulation microchips

The microfabrication and operation of the mechanostimulation microchips have been described previously in more detail.^31^ In short, on an oxidized Si wafer, an Au layer and a thin Cr adhesion layer were thermally evaporated. The photo‐patternable resin SU8 and electroactive polymer PPy were photolithographically patterned on the Au layer to form the different microactuators on the microchip (Figure 1A). Next, the Au (and Cr) was wet chemically etched to form the final electrode structure, and the wafer was diced into single mechanostimulation microchips (Figure 1B).

### 2.4 Cell stimulation

Cell stimulation experiments were performed at room temperature in DMEM without phenol red. Stock solutions of the different pharmacological agents were added to the cell culture dish comprising the cells to achieve the final concentrations. ATP, UTP, ADP, PPADS, apyrase (EC 3.6.1.5) and routine chemicals were acquired from Sigma‐Aldrich (St Louis, USA). Dilutions of stock solutions were prepared with deionized water (18.2 MΩ) before experiments. To test if the apyrase formulation had unspecific blocking effect not related to its enzymatic activity we tested if apyrase, which is a Ca^2+^‐dependent enzyme, had any blocking effect in Ca^2+^‐free media.

To perform mechanical stimulation, the microchip comprising the cells was mounted in a customized chamber in DMEM/F12 without phenol red. The mechanostimulation microchips were operated using a Gamry potentiostat Ref600 with Gamry PHE200 software. For Ca^2+^ imaging, a 300 seconds stimulation of −1.0 V was followed by a period of 60 seconds at 0.0 V against the Ag/AgCl reference electrode. More details about the stimulation can be found in.^31^

### 2.5 Construction of silicon‐based cell stretch chamber

Cell culture chambers, were fabricated by moulding silicon elastomer in an aluminium master form. Sylgard 184 Elastomer kit from DOW CORNING (USA) was used. Ratio of curing agent and base was 1:10. After pouring the mix into the master form, it was left in room temp until degassed and then cured in an oven at 70°C for 24 hours. The cured cell stretch chambers were cleaned by incubation in 70% ethanol and then in distilled water. Before use, they were autoclaved in 120°C for 20 minutes. The chamber was seated on a pneumatic system driven by a syringe pump as shown in Figure 1C. Infusing the syringe with a certain volume controls the degree of stretch applied to the chamber. We used an infusion of 1 mL to accomplish a degree of cell stretch that corresponded to a dislocation of approximately 20%.

### 2.6 Measurement of ATP and LDH release

In the series of stretch‐induced ATP release experiments, T24 cells were cultured in PDMS cell culture chambers coated with 2 μg/cm^2^ laminin (Sigma‐Aldrich) in the same condition as mentioned above. Samples were taken and placed on ice immediately before and after stretching. A luciferin‐luciferase‐based reaction kit (Molecular Probes) was used to analyse extracellular ATP release. As control samples, cell lysate was prepared by lysing T24 cells cultured in the stretch culture chambers by adding 0.1% Triton X‐100 in PBS for 10 minutes at room temperature. Samples were analysed in 96‐well plates. LDH release was also analysed using Pierce™ LDH Cytotoxicity Assay Kit acquired from ThermoFisher Scientific. Analysis was performed in 96‐well plates according to the suppliers’ instructions. ATP and LDH samples were analysed using a Spectra Max 190 plate reader (Molecular Devices).

### 2.7 Immunochemistry

For immunocytochemistry experiments, cells were cultured on coverslips. Prior to staining, cells were fixed in 4% formaldehyde for 10 minutes and then rinsed in PBS. Specimens were pre‐incubated in PBS containing 0.05% Triton X‐100 and 0.2% bovine serum albumin (BSA; both from Sigma‐Aldrich, St Louis, USA). Slides were then incubated with primary antibodies; guinea pig anti‐P2X3 (1:250; Novus Cat# NB100‐1658, RRID:AB_10001676, rabbit anti‐P2Y_6_ (1:50; H‐70; Santa Cruz Biotechnology Cat# sc‐20127, RRID: AB_2156250), diluted in PBS containing 0.05% Triton X‐100 and 0.2% BSA for 1 hour in a moist chamber at room temperature. After washing in PBS, the slides were incubated with secondary antibodies: goat anti‐rabbit antibody labelled with Cy 3 (1:500; Jackson ImmunoResearch Labs Cat# 111‐165‐144, RRID:AB_2338006) or goat anti‐guinea pig antibody labelled with Cy5 (1:500; Novus Cat# NB120‐6567, RRID:AB_790417), actin‐binding FITC‐phalloidin (1 μg/mL; Sigma‐Aldrich) and Hoechst 33258 (1:2000; Sigma‐Aldrich, 94403) for one hour at room temperature in a moist chamber. Slides were then rinsed in PBS before mounted with Dako Fluorescence Medium (DakoCytomation, Glostrup, Denmark) to prevent fading. Secondary antibody specificity was assessed by omitting the primary antibody. Human bladder tissue was used as positive controls. Collection of human bladder tissue was carried out in accordance with the Declaration of Helsinki and was approved by the Regional Ethics Committee at Karolinska University Hospital (Dnr. 2010/574‐32, 2009/1481‐32, 2008/1633‐31). All collected tissue was prepared as previously described.^28^ Immunofluorescence image acquisition was performed with a laser scanning confocal microscope Zeiss LSM 800. Image analysis was conducted using Image J (U. S. National Institutes of Health).

### 2.8 Statistics and data analysis

All data are presented as mean ± SEM. Student's paired and unpaired *t* tests were used for comparison of differences between two groups, in cases where the data were not normally distributed according to Shapiro‐Wilk test, Mann‐Whitney *U* test was used instead of *t* test. For comparisons between more than two groups, ANOVA with Bonferroni correction for multiple comparisons was used.

## 3 RESULTS

### 3.1 Changes in cytosolic Ca^2+^ concentration by mechanical stimulation and ATP

**Figure 2:**
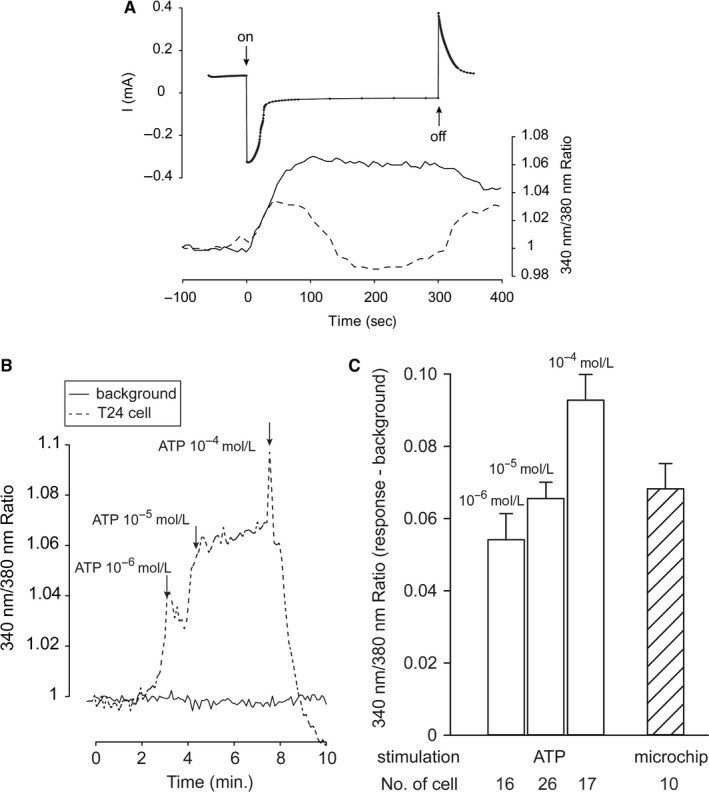
Ca^2+^ responses in T24 cells induced by mechanical stimulation using mechanochip and ATP. A, Upper tracing showing chronoamperometry scan curve by potentiostat. Lower tracing showing intracellular Ca^2+^ signalling increasing due to mechanical stimulation (solid line) induced by mechanochip. Dashed line shows responses from cell on control surface. Upper tracing and lower tracing share the same timescale. B, The original tracing of intracellular Ca^2+^ signalling increasing to accumulative application of ATP from 10^−6^ mol L^−1^ to 10^−4^ mol L^−1^. C, Comparisons of intracellular Ca^2+^ increase induced by 10^−6^ mol L^−1^ to 10^−4^ mol L^−1^ ATP and mechanical stimulation. The Ca^2+^ responses induced by microchip correspond to application of 10^−5^ mol L^−1^ ATP. Data were collected from several cells in different experiments and presented as mean ± SEM

T24 cells were cultured on mechanostimulation microchips based on the electromechanically active polymer polypyrrole (PPy) illustrated in Figure 1A,B. The mechanochip delivers a vertical stretch of approximately 1 μm to cells cultured on the active sites of the chip.^31^ When mechanical stimulation was applied to T24 cells, they responded with an increase in the intracellular Ca^2+^ concentration as can be seen from the lower tracing in Figure 2A. Cells cultured on the control PPy surface received the same electric stimulation (−1 V) and responded with peaks in cytosolic Ca^2+^ concentration coinciding with the changes of the electrical current. Next, ATP was used to stimulate the T24 cells. Accumulative application of ATP from 10^−6^ to 10^−4^ mol L^−1^ resulted in a similar increase in the intracellular Ca^2+^ concentration as seen with mechanical stimulation, as shown in Figure 2B. The amplitude of cytosolic Ca^2+^ increase seen with the mechanical stimulation corresponded to the effects of exogenous application of 10^−5^ mol L^−1^ ATP (Figure 2C).

**Figure 3:**
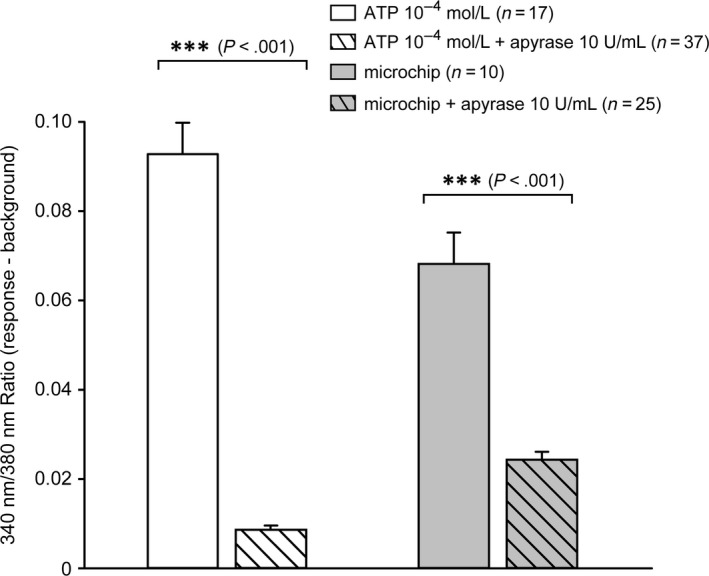
Summary of apyrase blocking microchip mechanical stimulation and 10^−4^ mol L^−1^ ATP‐induced Ca^2+^ responses in T24 cells. The addition of 10 U/mL apyrase blocked the effects of exogenous application of 10^−4^ mol L^−1^ ATP by 90% and effects of mechanical stimulation by around 65%. Data presented as mean ± SEM. ***denotes *P* < .001 by Student's unpaired *t* test. n denotes number of cells from different experiments

To investigate if the mechanisms that lead to the increase in intracellular Ca^+2^ concentration seen with both mechanical stimulation and ATP are connected, we inhibited the ATP‐induced cytosolic Ca^2+^ increase, using the ATP‐diphosphohydrolase apyrase. As shown in Figure 3, 10 U/mL apyrase blocked 90% of the increase in intracellular Ca^2+^ induced by 10^−4^ mol L^−1^ ATP. When mechanical stimulation was administered to cells on mechanochips, 10 U/mL apyrase blocked increase in cytosolic Ca^2+^ by more than 65%. The blocking effect of apyrase was significant for both ATP stimulation as well as mechanical stimulation.

### 3.2 ATP release induced stretching of cells

**Figure 4:**
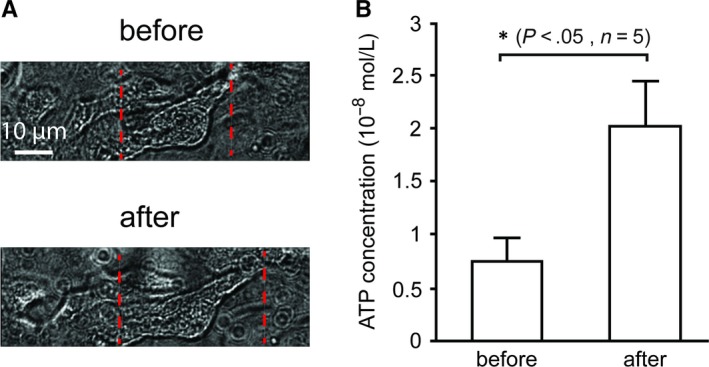
Measurements of ATP release increasing in T24 cells after stretch using PDMS cell culture chambers. A, Typical cell images before and after stretches. The attached T24 cell between two red dotted lines was elongated by about 20% after stretch. Scale bar = 10 μm. B, Summary of ATP release using luciferin‐luciferase reaction kit before and after stretches. Data presented as mean ± SEM. *denotes *P* < .05 by Student's paired *t* test, n denotes number of experiments

The blocking effect of apyrase on the increase in intracellular Ca^2+^ induced by mechanical stimulation suggests that release of ATP upon mechanical stimulation contributes to this response. To investigate if ATP is also released upon mechanical stimulation of a population of cells, we developed a stretch device (Figure 1C) for stretching of an entire population of cells as the mechanochip only activates single cells located on the active sites. Stretching of cells at a dislocation of approximately 20% (Figure 4A) resulted in a two‐ to threefold increase of the ATP concentration in the cell culture media as shown in Figure 4B. Cell damage due to stretching defined by lactate dehydrogenase release was 1.1% ± 0.3% (mean ± SEM, n = 5).

### 3.3 Characterization of ATP response

**Figure 5:**
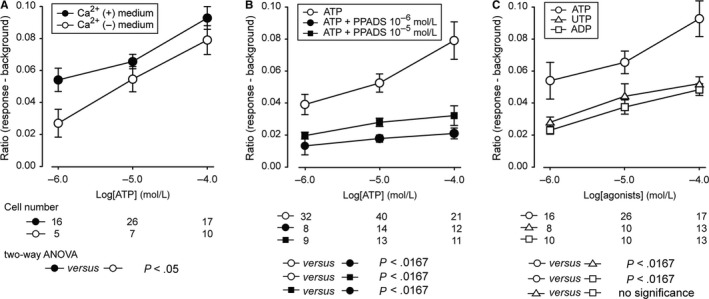
Investigations on purinergic P2X and P2Y receptors involved in the stretch‐related Ca^2+^ responses in T24 cells using pharmacological reagents. A, ATP 3‐dose‐response from 10^−6^ mol L^−1^ to 10^−4^ mol L^−1^ in media with and without calcium. B, ATP 3‐dose‐response from 10^−6^ mol L^−1^ to 10^−4^ mol L^−1^ in the presence and absence of non‐selective P2X antagonist PPADS 10^−6^ mol L^−1^ and 10^−5^ mol L^−1^. C, 3‐dose‐response from 10^−6^ mol L^−1^ to 10^−4^ mol L^−1^ generated by ATP and different P2Y receptor agonists UTP, ADP. Data were collected from several cells in different experiments and presented as mean ± SEM

To further understand the signalling pathways induced by stretching, the purinergic receptors involved in the ATP‐induced increase in cytosolic Ca^2+^ concentration were investigated. Receptors of the P2X family are ligand‐gated ion channels, and therefore, the increase in intracellular Ca^2+^ concentration mediated by the P2X receptors is dependent on extracellular Ca^2+^. P2Y receptors, on the other hand, are G‐protein‐coupled receptors that can induce release of Ca^2+^ from intracellular stores via the IP3 pathway.^32^ To differentiate between P2Y and P2X, we stimulated T24 cells with ATP in media containing Ca^2+^ as well as in Ca^2+^‐free media. ATP dose‐response curves from 10^−6^ to 10^−4 ^mol L^−1^ were generated, and the cells responded with a dose‐dependent increase in intracellular Ca^2+^ regardless if there were Ca^2+^ in the media or not (Figure 5A). However, the response was lower in the Ca^2+^‐free media; this difference was statistically significant. In Ca^2+^‐free media, addition of apyrase did not have a blocking effect.

The effect of the non‐selective purinergic blocker PPADS was tested in T24 cells (Figure 5B). PPADS at concentrations of 10^−6 ^mol L^−1^ and 10^−5 ^mol L^−1^ gave a statistically significant decrease in the intracellular Ca^2+^ response to ATP.

P2Y receptor agonists, UTP and ADP, were tested, and dose‐response curves from 10^−6^ to 10^−4 ^mol L^−1^ were generated. Addition of UTP as well as ADP led to an increase in the cytosolic Ca^2+^ concentration, which was slightly less than that seen with ATP (Figure 5C). Compared with purinergic receptors, agonists like carbachol, serotonin and histamine barely affected intracellular Ca^2+^ concentration in T24 cells up to concentrations of 10^−4^ mol L^−1^ (Figures S1 and S2).

### 3.4 Expression of P2 receptors in T24 cells

**Figure 6:**
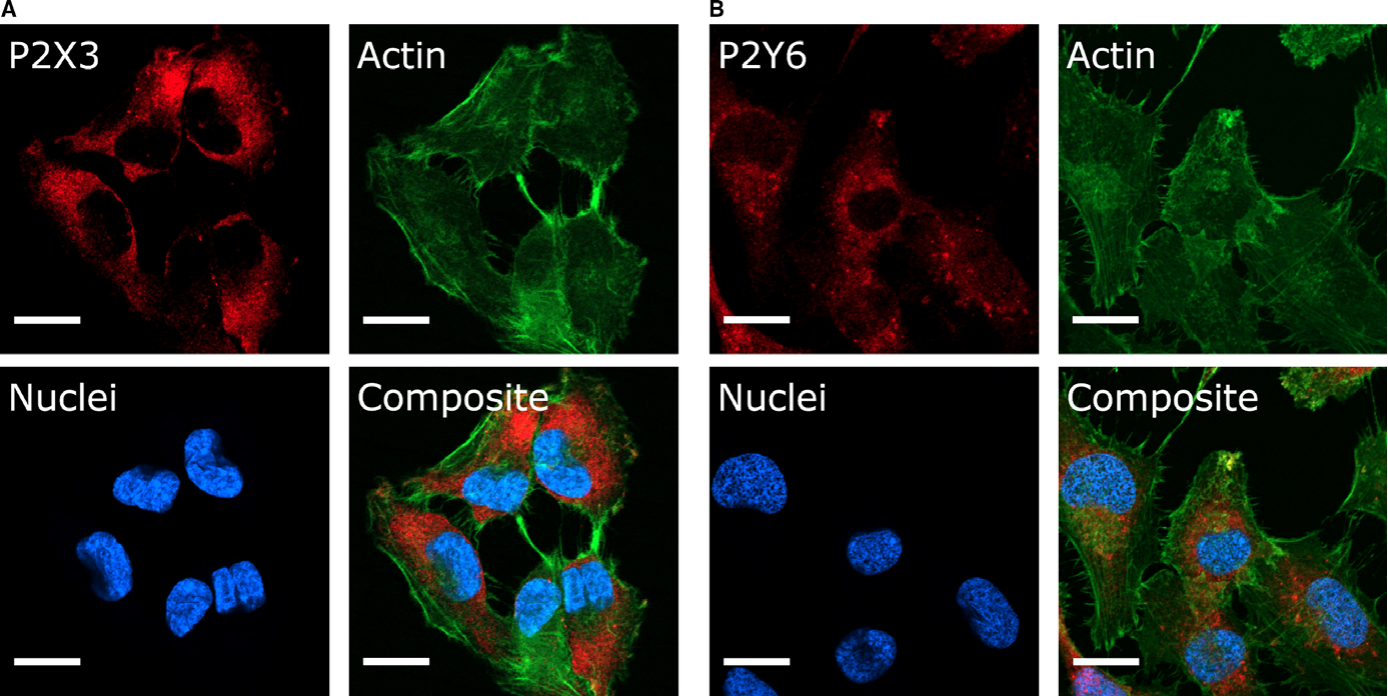
Fluorescence micrographs depicting immunoreactivity to P2 receptors in T24 cells. (A) P2X3, (B) P2Y_6_. P2 receptor subunit (red), cytoskeletal actin fibres (green) and cell nuclei (blue); scale bar = 15 μm

Polyclonal antibodies against the G‐protein‐coupled receptor P2Y_6_ as well as the ion channel P2X3 were used to identify these receptors on the T24 cells using immunocytochemistry. These receptors were chosen because they have been shown to be present in urothelial cells.^26^, ^28^, ^33^ Immunoreactivity was seen with antibodies against both P2 receptors, see Figure 6. Hoechst 33258 was used to visualize the cell nucleus, and the actin‐binding toxin phalloidin conjugated to FITC was used to stain actin to delineate the contours of the cells. Immunoreactivity was present throughout most of the cell with the most intense staining just outside the nucleus. Indicating a significant part of the immunoreactivity may be in the cytoplasm. In negative controls where the primary antibody was omitted, no immunoreactivity was seen (data not shown). Human bladder tissue was used as positive controls (Figure S3).

## 4 DISCUSSION

In this study, we set out to test the hypothesis that ATP is important for the changes in cytosolic Ca^2+^ concentration induced by mechanical stimulation of T24 urothelial cells. To accomplish this, we introduced new tools, a mechanostimulation microchip and a silicon‐based cell stretcher, which we believe are of value for anyone interested in cellular mechanotransduction. We show that ATP is released from T24 cells upon mechanical stimulation; by blocking extracellular ATP, we could see a significant decrease in the response induced by mechanical stimulation. In addition, we showed that T24 cells express different subtypes of purinergic receptors, both from the families of ionotropic P2X and metabotropic P2Y.

Many studies have shown that ATP is released from urothelial cells upon mechanical stimulation.^10^, ^34^, ^35^ Several reports also stress the importance of ATP as a non‐adrenergic, non‐cholinergic transmitter for signalling between the bladder and the central nervous system.^5^, ^36^, ^37^, ^38^ This crosstalk where information about mechanical stimulation is relayed by purinergic signalling of sensory and parasympathetic nerves is well described in terms of mechanosensory transduction. The focus of this paper has been limited towards the role of auto‐ and paracrine purinergic signalling as a mechanism involved in cellular mechanotransduction. The exact role of ATP in cellular mechanotransduction in the urinary bladder is unclear. Some reports show an increase in intracellular Ca^2+^ concentration before release of ATP, indicating that the mechanosensing is initiated by mechanosensitive, possibly Ca^2+^‐conducting receptors.^39^, ^40^, ^41^ These findings suggest that the release of ATP is an active secretion that requires an increase in intracellular Ca^2+^ concentration to take place. Several mechanisms have been proposed for the release of ATP, for example electrodiffusional translocation via connexin‐ and pannexin‐containing hemichannels and voltage gated‐dependent anion channels, as well as facilitated diffusion by nucleotide‐specific ATP‐binding cassette (ABC) transporters and vesicle‐mediated release.^42^ For urothelial cells, there is compelling evidence that hemichannels is the more important.^25^, ^26^

Based on the results from this study, we suggest that the release of extracellular ATP and subsequent auto‐ and paracrine signalling are important factors for the initiation of a change in the cytosolic Ca^2+^ concentration due to mechanical stimulation in T24 cells.

The increase in intracellular Ca^2+^ concentration that we observe upon mechanical stretching is of the same magnitude as that obtained by adding 10^−5^ mol L^−1^ of ATP. This is in line with other reports where concentrations of 10^−4^‐10^−5^ mol L^−1^ of ATP were used to induce changes in cytosolic Ca^2+^ concentration in cells.^41^, ^43^ The mechanical stimulation is accomplished with an electrically driven mechanochip. The chip contains control areas where cells are exposed to the potential differences and currents that drive the actuators, but without the mechanical stimulation. The cells on the control areas show fluctuations in the intracellular Ca^2+^ concentration that coincide with the ion fluxes associated with the potential changes. We have seen these fluctuations previously and are currently investigating the exact nature of this phenomenon. By comparing the response of the stretched cells with the non‐stretched but electrochemically addressed cells, we can subtract the latter effect. Also, the mechanical expansion profile (expansion vs time) of the actuators is congruent with the response of the stretched cells, thus further indicating that the response is mechanically induced. The response we see with the mechanochip is long‐lasting and continues past the point where the potential is returned to zero. We think this lasting response is due to the fact that the PPy remains expanded, that is the strain on the cells remains even after the potential is returned to zero. We are currently developing a second generation of mechanochips where the strain will be reversible so that we can investigate the whole cycle of stretch and release on the cells. More detailed technical specifications of the mechanochip can be found in Svennersten et al^31^

Furthermore, we observed that we could significantly decrease the response induced by either ATP or mechanical stimulation, using apyrase. However, the blocking effect was stronger for ATP‐induced stimulation than for mechanically induced stimulation. This could be due to the fact that urothelial cells express many other mechanosensitive receptors that may contribute to the increase in intracellular Ca^2+^ concentration and that the autocrine ATP signalling works rather like an amplifier for the increase in the cytosolic Ca^2+^ concentration.^44^

We suggested that the significant blocking of the response induced by mechanical stimulation using apyrase suggested that the mechanical stimulation indeed resulted in release of ATP and subsequent autocrine signalling. To corroborate this finding, we measured ATP release upon mechanical stretching of the cells. Because the mechanochips only stimulate single cells, the released quantities of ATP from a single cell were too small to be measured using the luciferin‐luciferase assay. Therefore, we devised an alternative stretching tool using silicone elastomer that could stretch a whole population of cells, thus ensuring the release of a measureable amount of ATP. We observed that this stretching resulted in a significant release of ATP, which is in accordance to what others have found in bladder tissue experiments.^39^, ^40^

As there are two major families of ATP receptors, the ionotropic P2X and the metabotropic P2Y, we wanted to investigate if both of these contributed to the response that we measured or whether one family of receptor was more dominant than the other. Our first approach was to perform the experiments in Ca^2+^‐free media, as P2X receptors depend on extracellular Ca^2+^. The responses seen in Ca^2+^‐free media were slightly smaller than those seen in media containing Ca^2+^, this difference was statistically significant. Upon testing agonists such as ADP and UTP, which activate P2Y rather than P2X, responses were slightly smaller compared to ATP. This finding suggests that both P2Y and P2X receptors are expressed by T24 urothelial cells and that they have a significant contribution to the Ca^2+^ response seen upon stimulation with ATP. The role of P2Y receptors is important as a significant part of purinergic auto‐ and paracrine signalling in the bladder is composed of the metabolite ADP likely due to NTPDases expressed in the urothelium.^34^, ^45^, ^46^, ^47^ We also tested other pharmacological substances such as carbachol, serotonin and histamine. The effect of these substances was barely measurable. In the case of carbachol, this is interesting since activation of nicotinic as well as muscarinic acetylcholine receptors has been shown to trigger release of ATP from urothelial cells.^48^, ^49^ However, those studies did not use carbachol. Sui et al showed that carbachol can trigger release of ATP from urothelial cells but without significant increase in intracellular Ca^2+^.^50^ Therefore, it would be interesting in future studies to test if carbachol can trigger Ca^2+^‐independent ATP release in T24 cells as well.

From the former findings, we conclude that P2Y receptors make a major contribution to the observed response. To further verify this, we tested the non‐selective purinergic receptor blocker PPADS, which is primarily described as a P2X antagonist at moderate concentrations. PPADS had a partial inhibitory effect, strengthening the conclusion that the observed responses comprised a large component of P2Y activity. However, some caution should be taken in making conclusions based on the PPADS results, as PPADS is reported to antagonize both P2X receptors at moderate concentrations^51^ and P2Y receptors at high concentrations.^52^ We saw relatively large blocking effect using PPADS at moderate concentrations compared to the effect of using Ca^2+^‐free media, indicating that even at the concentration of PPADS that we use, there is some effect also on P2Y receptors. PPADS also has dual actions not only as an antagonist of P2‐purinoceptor but also as an ecto‐ATPase inhibitor.^53^

With compelling pharmacological evidence for P2 receptor signalling, we wanted to investigate the expression of P2 receptors using immunochemistry methods. Previous studies had shown no effect on cAMP upon activation of P2Y in T24 cells making it likely that P2Y receptors in T24 cells are G_q_‐coupled rather than G_s/i‐_coupled.^54^ Therefore, we chose to look for the expression of a P2Y receptor that was known to be G_q_‐coupled and also known to be found in urothelial cells, such as P2Y_6_.^32^ We also investigated the expression of P2X3 receptors based on previous immunohistological findings from the human urothelium.^28^ We found that in the T24 cells, there was immunoreactivity for both P2Y_6_ and P2X3, which is not surprising as these are previously shown in urothelial cells.^24^, ^26^, ^28^, ^33^ However, regarding P2Y_6_ our functional data neither support nor oppose the presence of this subtype as it is mainly activated by UDP, only to a lesser extent by UTP and barely by ADP and ATP.^32^ With this in mind, we think that it is very likely that also other subtypes are present. The pattern of the immunoreactivity for P2X3 and P2Y_6_ seen in this paper indicates that a significant part of receptors is located intracellularly; this might indicate that the cells have a continuous overexpression of these receptors which would not be surprising as the cells are derived from a urothelial cancer. Worth mentioning is that the distribution of immunoreactivity is similar to that of others who have stained P2Y receptors in cell cultures.^55^ As a counterstain, we chose to use actin‐binding phalloidin rather than antibodies against cytokeratins. The main reason for this was to avoid possible cross‐reaction that might occur when using several antibodies simultaneously. Also staining of actin serves the purpose well to give clear delineation of the cells borders.

The work conducted in this paper is based on the T24 urothelial cell line, which is isolated from an urothelial carcinoma. This is a major limitation, and one should be careful not to extrapolate our results to mechanisms present in the healthy urinary bladder. However, the T24 is a robust cell line suitable for conducting this study where we introduce new tools to study mechanotransduction on a cellular level. In this work, we have employed new microphysiological systems for mechanical stimulation, a mechanostimulation microchip and a silicon‐based cell stretcher, and established basic principles of mechanotransduction on a cellular level. Our next step is to apply these methods and to test our hypothesis on human primary cells and tissues. We also hope that these new tools might serve as inspiration for our peers in the field of mechanotransduction.

Conclusively, T24 cells express P2Y and P2X receptors, both of which contribute to the change in cytosolic Ca^2+^ concentration seen upon mechanical stimulation. However, P2Y seems to be the dominating contributor to this response. The autocrine ATP signalling associated with mechanotransduction in urothelial cells contributes to a major part of the mechanotransduction‐mediated changes in cytosolic Ca^2+^ concentration in T24 cells. The mechanostimulation microchips and silicon‐based cell stretcher are effective, new tools to study mechanostimulation pathways at the cellular level.

## AUTHOR CONTRIBUTION

NNG, KS and EWHJ conceived and designed the study. NNG and KS performed calcium and stretch experiments. EWHJ designed and fabricated microchips. NS and KS performed immunochemistry experiments. NNG, NS, EWHJ and KS prepared figures. NNG, EWHJ and KS wrote the first draft of the manuscript. KHG contributed control tissue and evaluated immunohistochemistry data. All authors contributed with editing of the paper and approved the final version.

## CONFLICT OF INTEREST

The authors declare that they have no competing interests.
