# Supplementary figures and images for: The role of ATP signalling in response to mechanical stimulation studied in T24 cells using new microphysiological tools
Source: J Cell Mol Med. 2018 Feb 1;22(4):2319–28. doi: 10.1111/jcmm.13520 (PMC5867107; doi:10.1111/jcmm.13520)

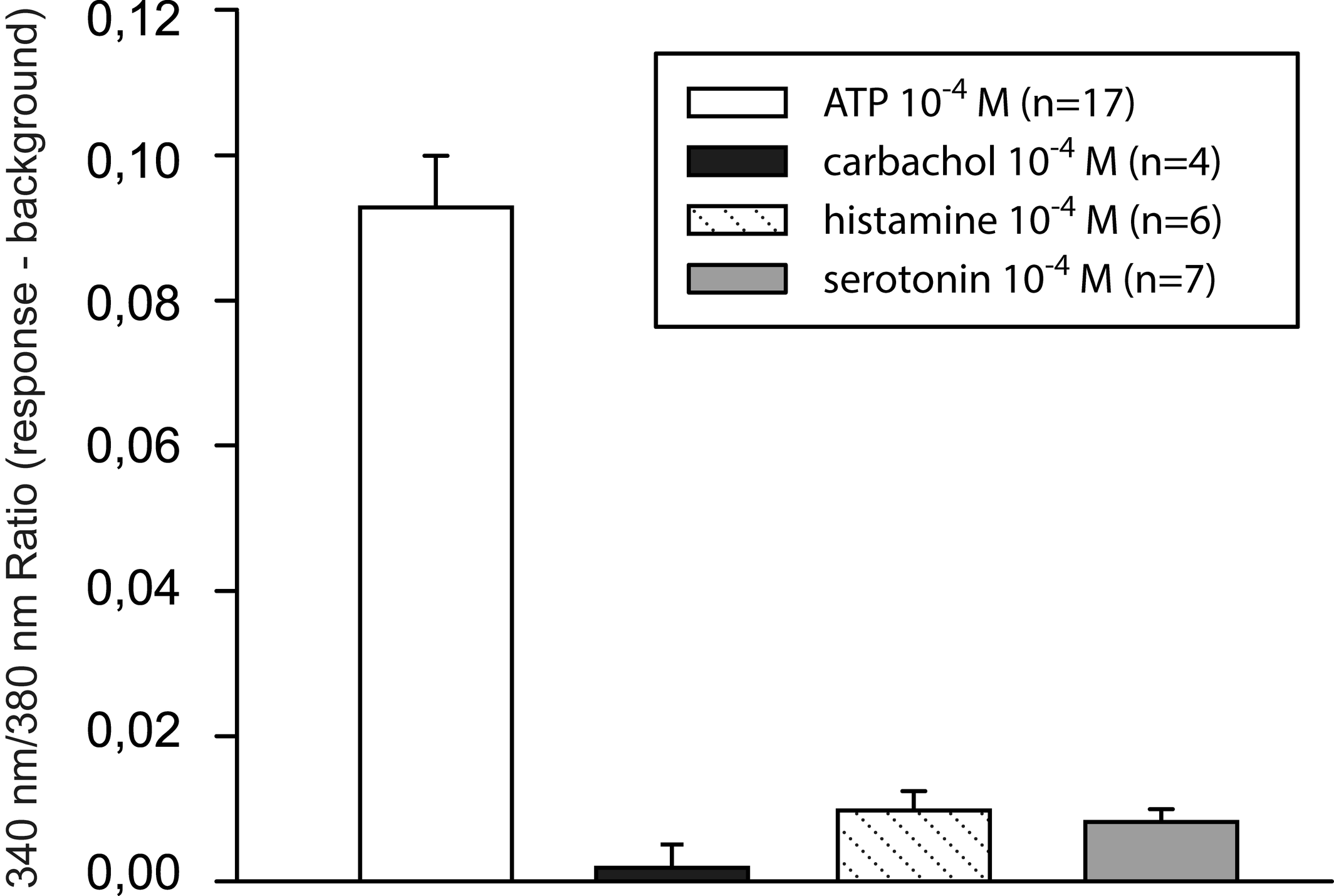

Supplement: Supplementary file 1 [file JCMM-22-2319-s001.tif]

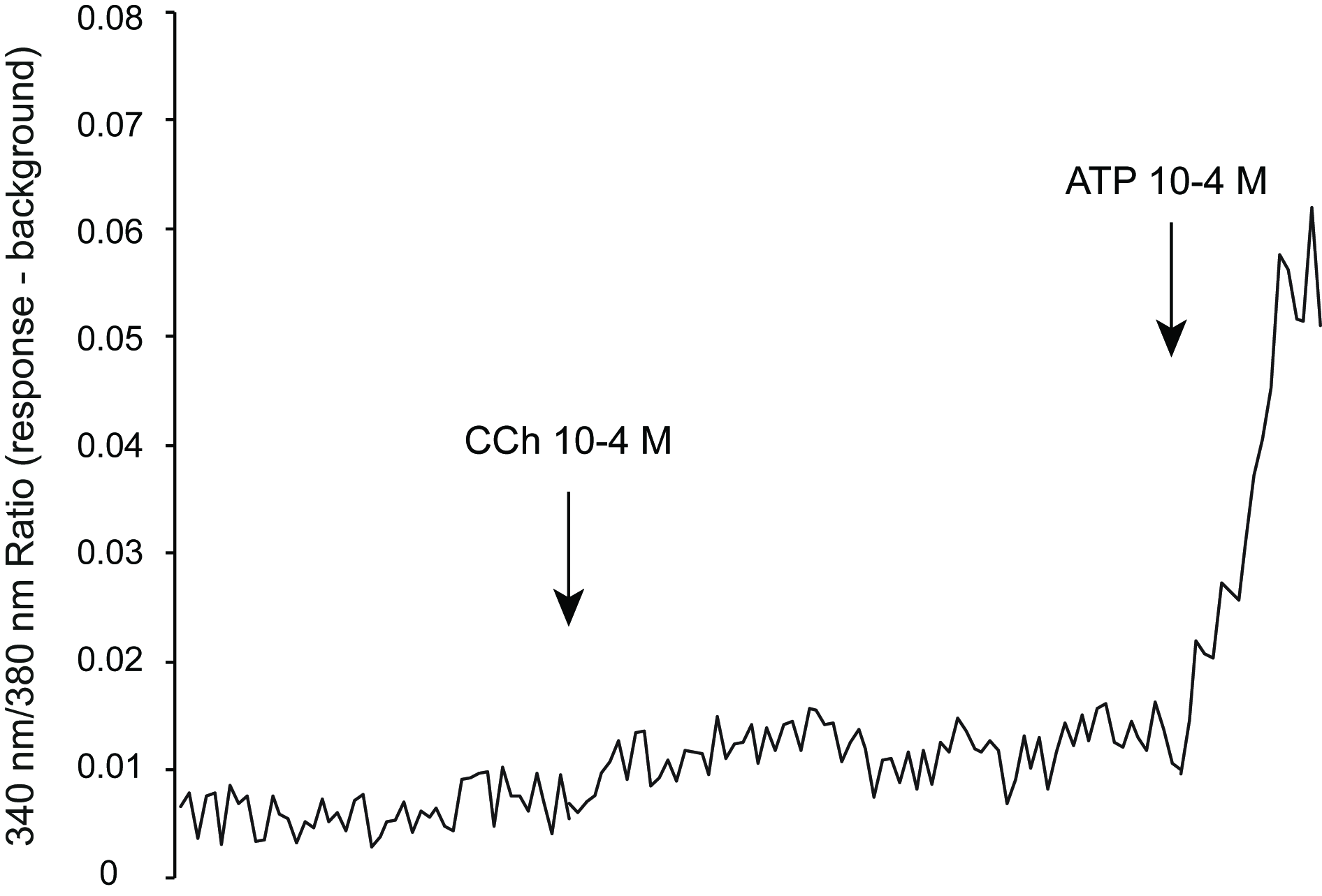

Supplement: Supplementary file 2 [file JCMM-22-2319-s002.tif]

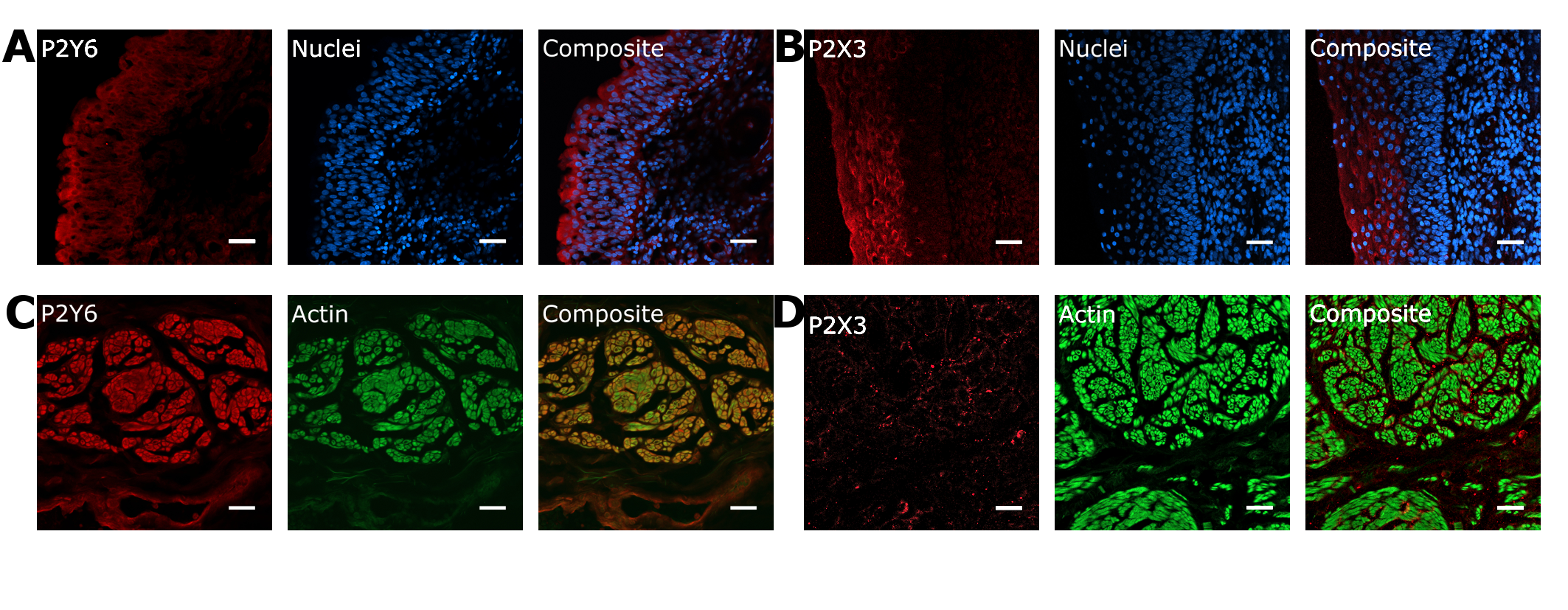

Supplement: Supplementary file 3 [file JCMM-22-2319-s003.tif]
